# Development and validation of the Chinese version of the perceived partner responsiveness scale (C-PPRS)

**DOI:** 10.1186/s40359-022-00865-x

**Published:** 2022-06-20

**Authors:** Xinyi Zhou, Yaochun Cai, Wanyi Huang, Qishan Chen

**Affiliations:** 1 Philosophy and Social Science Laboratory of Reading and Development in Children and Adolescents (South China Normal University), Ministry of Education, Guangzhou, 510631 China; 2 School of Psychology, South China Normal University, Guangzhou, 510631 China; 3 State Key Laboratory of Cognitive Neuroscience and Learning & IDG/McGovern Institute for Brain Research, Beijing Normal University, Beijing, 100875 China; 4 Department of Education, Institute of Educational Psychology and School Counseling, Beijing Normal University, Beijing, 100875 China

**Keywords:** Perceived partner responsiveness, Scale development, Chinese version, Validity, Reliability

## Abstract

**Background:**

Perceived partner responsiveness (PPR) refers to the belief that the relational partner knows and is sensitive and supportive. Instead of translating the English version of the Perceived Partner Responsiveness Scale (PPRS) into Chinese, this study aimed to construct and analyze the psychometric properties of the Chinese version of the Perceived Partner Responsiveness Scale (C-PPRS). On the one hand, some words in the original scale are inappropriate for the Chinese due to cultural differences. On the other hand, we intended the scale to apply just to persons in romantic relationships, not to friends or roommates.

**Method:**

We conducted two studies. In the first study, 441 participants who completed the C-PPRS were randomly divided into two samples for exploratory factor analysis and confirmatory factor analysis. Concurrent validity was assessed in a group of 224 participants who completed the C-PPRS and the Quality of Relationship Index in the second study.

**Results:**

The results indicated that the four-factor model (understanding, intimacy, acceptance, and trust) was a feasible representation of the C-PPRS factor structure (*χ*^2^/*df* = 2.27, CFI = 0.94, TLI = 0.93, RMSEA = 0.08, SRMR = 0.05) and had robust internal consistency reliability (alpha = 0.90) and concurrent validity (moderately correlated with the Quality of Relationship Index, *r* = 0.66, *p* < 0.001).

**Conclusion:**

PPR is a concept to understand the psychological manifestations of a person who believes that his or her partner is concerned with core characteristics of the self. The C-PPRS has good psychometric characteristics to evaluate such manifestations and can be applied to future intimacy research.

**Supplementary Information:**

The online version contains supplementary material available at 10.1186/s40359-022-00865-x.

## Introduction

Being in a romantic relationship or being in love with someone is one of the most common and important feelings that humans may experience in their lifetime. Many studies have shown that healthy romantic relationships improve our physical and mental health and that the quality of romantic relationships is highly correlated with people’s blood pressure, life satisfaction, and so on [1, 2]. Perceived partner responsiveness (PPR) plays a crucial role in the interaction between an individual and his or her partner. PPR means one’s belief that his or her partner is concerned about core features of self, for example, the goals, values, traits, abilities, and attributes [3].

As an important construct in intimacy research, PPR is considered the “central organizing structure” of relationship research [3]. PPR can not only promote an individual’s psychological health and relationship quality or relational satisfaction with a partner by increasing the individual’s intellectual humility and encouraging their emotional expression but also explain the relationship among self-disclosure, partner disclosure, and intimacy through its mediating effect [4–7].

Regarding the measurement instruments for PPR, an earlier unpublished English version of the Perceived Partner Responsiveness Scale (PPRS) was developed by Reis, which contained 18 items [8]. However, after some minor modifications, a 12-item unidimensional PPRS with better validity was published in 2011 [9]. Meanwhile, Kubacka et al. developed a PPRS for married couples based on Reis and Shaver’s model with 18 questions describing PPR in terms of acceptance, understanding, and care [10, 11]. Recently, based on the interpersonal process model of intimacy, Reis et al. modified the original version and developed a new scale that contains two dimensions (understanding and validation) with 18 questions [12]. The above scales were developed based on Western cultural situations. Given the cultural differences, Chinese people may differ from Western cultural subjects in their feelings and responses to intimacy. The purpose of the present study was to develop and examine the psychometric characteristics of the Chinese version of the Perceived Partner Responsiveness Scale (C-PPRS).

### Research hypotheses

Initially, we tried to translate the English version of the PPRS [9, 12] into Chinese. However, we decided to develop a new version rather than use the translated version in the end. The first reason is cultural differences. Due to the differences between China and Western countries, some words in the original scale were difficult to translate and understand exactly. For example, the words emphasized in quotation marks in the original item, “the same wavelength”, “gets the facts right”, and “real”. Another example, when translated into Chinese, the item “usually seems to focus on the ‘best side’ of me” turns out to be very strange, with a negative grumbling mood. Therefore, we conducted a pilot study and collected feedback from the participants. The participants informed us that they found some words inappropriate enough for Chinese readers. They felt confused about the words and had to consider them for a long time when making responses. Furthermore, some of the original items are ambiguous in Chinese, such as “listens to me”, which can mean either that the partner listens to the actor and gives some response or that the actor controls and manages the partner. For many couples or people in a relationship in China, “listens to me” usually means the second meaning, but the original scale is intended to measure the first meaning.

Besides, the differences in measurement subjects were also considered before we decided to develop a new version of PPRS. All previous PPRS have been adapted to measure not only people in romantic relationships but also people in friendships and/or roommate relationships. However, the subjects of our study were only people in romantic relationships. Given the above reasons, a psychometrically robust scale that assesses PPR perfectly applicable for Chinese and is also able to show features of romance is warranted.

Finally, we developed the research hypothesis regarding the components of PPR according to the theoretical framework of the previous English version and the interview results of the pilot study. PPR was derived from the intimacy model proposed by Reis [3, 11]. According to this model, if an individual wants to feel intimacy from the partner in an intimate relationship, he or she needs to perceive the other person’s understanding, validation, and caring. Understanding means that the partner accurately perceives his or her needs and feelings, validation means that the partner respects and appreciates his or her inner self, and caring means that the partner will meet his or her essential needs and care about his or her emotions [3, 11]. Further, Reis expanded this structure to four aspects and gave specific descriptions [13], (1) the relational partner understands and is responsive to the self’s core attributes in social interactions; (2) the individual feels intimacy and warmth from the relational partner; (3) the relational partner accepts and offers to help address the individual’s needs in a supportive manner, and (4) the relational partner understands, trusts and appreciates what is important to the individual.

Based on the above elaboration, we develop the following hypothesis:

#### Hypothesis 1.

C-PPRS contains four factors (understanding, intimacy, acceptance and trust).

This newly developed scale, C-PPRS, was designed by creating 32 items. We deleted some items that were ambiguous or difficult for Chinese participants to understand from the English version of the PPRS [12], such as “sees the ‘real’ me” and “is on ‘the same wavelength’ with me”. We also created some new items to adapt to the Chinese culture and made it more romantic.

Given the significant changes in the construct structure and items, as recommended in the psychometrics literature, exploratory factor analysis (EFA) and confirmatory factor analysis (CFA) were conducted via two different sets of analyses using different samples in study 1 [14].

Studies have identified responsiveness as the active ingredient that underlies many essential qualities that define satisfying, healthy relationships, lies at the core of promoting well-being in romantic relationships, and contributes to closeness, intimacy, and the quality of the relationship [3, 15, 16]. Given that PPR is a strong predictor of relationship quality, this study examines the validity of C-PPRS using the quality of the relationship as the criterion. Preliminary evidence of concurrent validity was also reported in study 2.

This study proposes the following hypotheses:

#### Hypothesis 2

C-PPRS has good structural validity, reliability, and concurrent validity.

## Study 1

### Method

#### Participants

In this study, participants were recruited through an online data collection platform (i.e., https://www.wjx.cn). A total of 499 subjects completed the online questionnaire. Participants who were not married or not in a romantic relationship and those who completed the questionnaire within 60 s or received incorrect answers to all lie detection questions (58 participants) were excluded, and 441 valid questionnaires were obtained for an effective rate of 88.38%. The participants’ demographic information is shown in Table 1. The participants were all adults and in a romantic relationship when completing the questionnaire. The average age of the participants was 26.84 (*SD* = 8.34). Of the 441 participants, 25.2% were male, and 74.8% were female. 225 participants (51.0%) were married, and 216 participants (49.0%) were currently in a relationship. Their average duration of the relationship was 5.16 years (*SD* = 6. 55).

**Table 1:** Sample characteristics

Variables	Study 1 (N = 441)		Study 2 (N = 224)	
	Frequency	Proportion	Frequency	Proportion
Gender				
Male	111	25.2%	49	21.9%
Female	330	74.8%	175	78.1%
Total	441	100%	224	100%
Marital status				
In love	216	49.0%	198	88.4%
Married	225	51.0%	26	11.6%
Total	441	100%	224	100%
Age				
18–25	269	61.0%	178	79.5%
26–30	66	15.0%	26	11.6%
31–40	74	16.8%	11	4.9%
41–50	21	4.8%	7	3.1%
51–60	10	2.2%	2	0.9%
> 60	1	0.2%	–	–
Total	441	100%	224	100%
Duration of the relationship				
(0,1]	119	27.0%	90	40.2%
(1,5]	201	45.6%	108	48.2%
(5,10]	64	14.5%	15	6.7%
(10,20]	34	7.7%	6	2.7%
(20,30]	20	4.5%	5	2.2%
> 30	3	0.7%	–	–
Total	441	100%	224	100%

#### Procedure

The 12-item English version of the PPRS was translated to Chinese through backward and forward translations by two independent bilingual experts using the parallel blind technique [17]. According to the feedback of participants in the pilot study, some items were confusing or meant nearly the same as others in Chinese culture. Therefore, we developed a 32-item C-PPRS by reference to the definition of PPR, previous PPRS, and participants’ suggestions. The 32-item C-PPRS contained four factors named understanding, intimacy, acceptance, and trust. There were eight items in each of them.

#### Measures

*Demographic Information* The Demographic questionnaire was used to collect the demographic characteristics for the sample. It included age, gender, marital status (married or in a romantic relationship), and duration of the relationship.

*The Chinese Version of the Perceived Partner Responsiveness Scale *(*C-PPRS*) The initial C-PPRS (An additional file shows this in more detail, see Additional file 1) consisted of four factors, understanding (e.g., “My partner understands what I prefer or hate”), intimacy (e.g., “My partner often expresses his or her love to me”), acceptance (e.g., “My partner embraces my flaws”), and trust (e.g., “My partner believes that I am good and reliable”). Each factor was composed of eight items which were rated on a 5-point Likert scale (1 = totally disagree, 5 = totally agree).

#### Data analysis

We used SPSS 24.0 to conduct item analysis, EFA, and reliability analysis. And we used Mplus 7.4 to conduct CFA.

Item analysis was performed by analyzing discrimination, item-factor correlation, factor-total and item-total correlation. Based on guidelines for validating a newly developed measurement [18], we conducted EFA to explore the factor structure of the C-PPRS, followed by CFA to evaluate whether all factors with scale items of our hypothesis could be distinguished empirically. To achieve this cross-validation, we randomly divided the sample (N = 441) into roughly two equal sub-samples: Sample A (N = 221) was used to perform EFA, and Sample B (N = 220) was for CFA.

### Results

#### Preliminary analysis

Based on the distribution of average scores about C-PPRS, the top and the bottom 27% of the sample (N = 441) were selected into high (N = 119) and low PPR groups (N = 119), respectively. Items’ scores of the two groups were used in discrimination analysis by checking if there were significant differences between the two groups in each item. Results of the independent sample *t*-test indicated that there were significant differences in the scores of 32 items between the two groups (*t* values ranging from 8.95 to 17.00, *p* < 0.001). In addition, we used *Pearson* correlation to analyze item-factor correlation (correlation between the score of one item and the average score of its factor) and factor-total correlation (correlation between the average score of one factor and the average score of the whole scale). Item-factor correlations were all significant, with *r* values ranging from 0.51 to 0.85, *p* < 0.001. Factor-total correlations were also all significant, with *r* values ranging from 0.84 to 0.86, *p* < 0.001. We then used the same method to analyze item-total correlation (correlation between the score of one item and the average score of the whole scale). Item-total correlations were all significant, with *r* values ranging from 0.40 to 0.73, *p* < 0.001.

#### Exploratory factor analysis

We conducted EFA to 32 items of C-PPRS with SPSS 24.0. Kaiser–Meyer–Olkin (KMO) measure of sampling adequacy was 0.91, and the result of Bartlett’s test of sphericity was *χ*^2^_(496)_ = 4025.65, *p* < 0.001, demonstrating the data could be used for EFA. Due to the fact that the four factors are correlated with each other, Promax rotation analysis was performed in the study, combined with analyzing the scree plot. In item selection, factor loadings of 0.40 or higher were considered meaningful. In addition, items loaded on an unintended factor or simultaneously more than one factor were excluded. As a result, 17 items were deleted after the first EFA. Then, we conducted the EFA again on the remaining 15 items to ensure that all of them had satisfactory factor loadings (see Table 2 for final items and their factor loadings). The result showed that the four factors accounted for 68.53% of the total variance. The eigenvalues of four factors were 1.21 (understanding, consisting of 4 items), 6.43 (intimacy, consisting of 3 items), 1.04 (acceptance, consisting of 4 items), and 1.61 (trust, consisting of 4 items), respectively. All items’ loadings on the corresponding factors ranged from 0.60 to 0.93.

**Table 2:** Items and factor loadings for four factors of the Chinese version of the Perceived Partner Responsiveness Scale

Item	Understanding	Intimacy	Acceptance	Trust
U6 My partner is usually clear about what my decision is based on	**0.83**	–0.13	0.01	0.08
U4 My partner understands what I prefer or hate	**0.80**	–0.13	0.13	0.02
U2 My partner understands what kind of life I want	**0.75**	0.21	–0.12	–0.02
U3 My partner can stand on my feet and understand my feeling	**0.65**	0.24	0.03	–0.04
I2 My partner often expresses how much he or she misses me	0.02	**0.93**	0.01	–0.03
I4 My partner often expresses his or her love to me	–0.07	**0.88**	0.02	0.03
I3 When my partner is not by my side, he or she will tell me that he or she misses me	0.06	**0.86**	0.01	0.01
A3 My partner seldom blames me blindly for my mistakes	–0.03	0.09	**0.90**	–0.16
A2 My partner thinks that I don’t need to make changes for him or her	0.08	–0.15	**0.77**	–0.03
A4 My partner embraces my flaws	0.16	0.06	**0.69**	0.08
A1 My partner accepts my bad side	–0.16	0.12	**0.60**	0.25
T1 My partner thinks that I am responsible for our relationship and family	0.05	-0.17	0.01	**0.88**
T4 My partner considers that our relationship is strong	0.09	0.12	–0.20	**0.80**
T2 My partner believes that I am good and reliable	0.001	–0.06	0.19	**0.75**
T8 My partner always believes me when I am misunderstood by others	–0.07	0.17	0.01	**0.74**

Factor loadings of each item on the assumed main factor and other factors are reported. Values are in bold if factor loadings are equal to or above 0.40

#### Confirmatory factor analysis

**Fig. 1:**
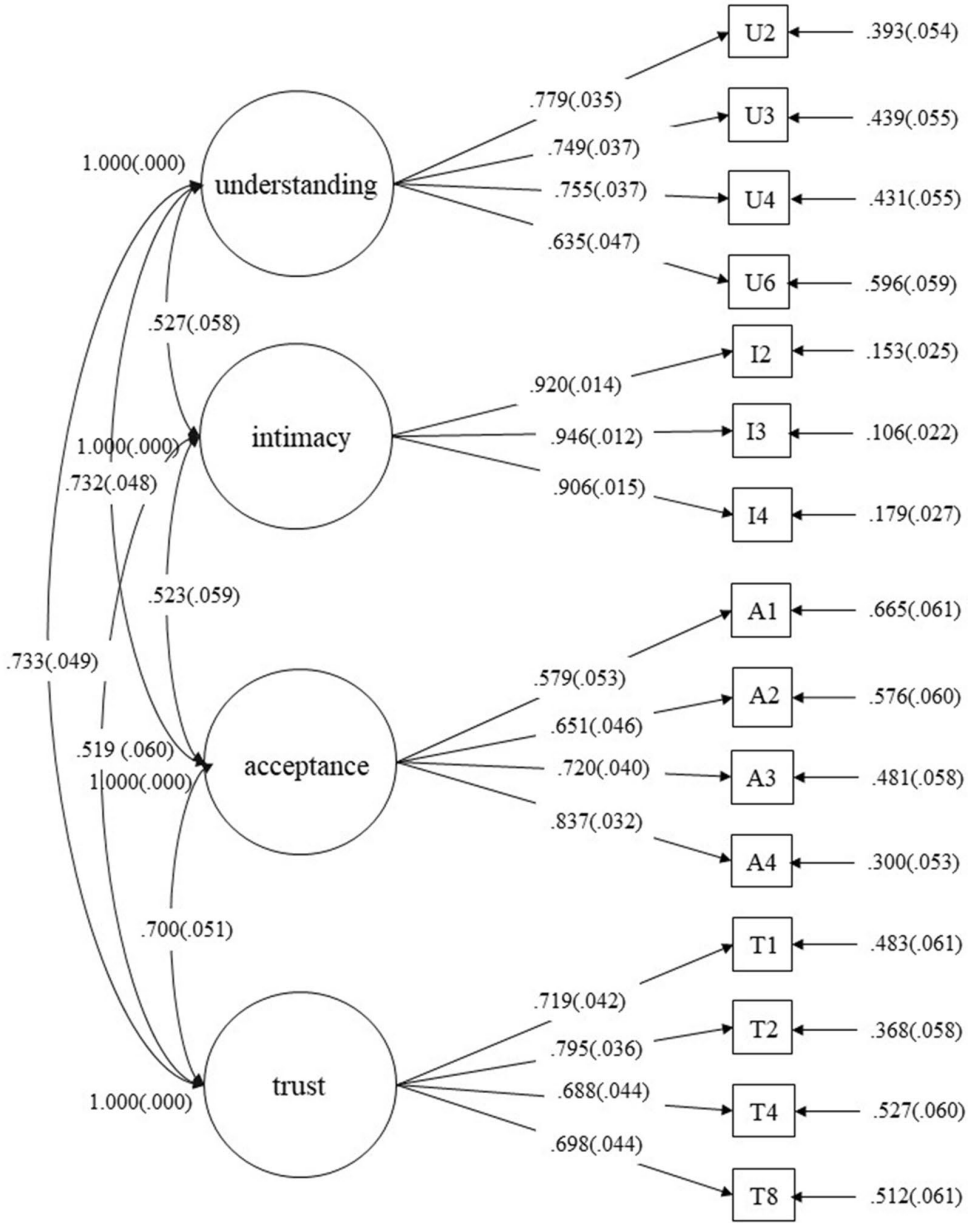
Path diagram from the CFA. *Note*: The standardized factor loadings with measurement error terms are reported

We conducted CFA to further assess the fitness of the measurement model using Sample B. Maximum likelihood estimation was performed to determine the standard errors for the parameter estimates. The model fit was considered acceptable when the comparative fit index (CFI) and Tucker-Lewis index (TLI) values were at or above 0.90, with root mean square error of approximation (RMSEA) and standardized root mean square residual (SRMR) at or below 0.08 [19]. We compared Model I with a single latent factor with Model II with four latent factors (i.e., understanding, intimacy, acceptance, and trust). The results showed that all indicators of Model I fall into the unacceptable range, χ^2^ = 761.48, *χ*^2^/*df* = 8.46, CFI = 0.64, TLI = 0.58, RMSEA = 0.18, SRMR = 0.10, however, the goodness-of-fit indices of Model II fitted the data well, χ^2^ = 190.3, *χ*^2^/*df* = 2.27, CFI = 0.94, TLI = 0.93, RMSEA = 0.08, SRMR = 0.05. The results further validated the four-factor structure of the C-PPRS. The loadings of 4 items in understanding, 3 in intimacy, 4 in acceptance, and 4 in trust were respectively from 0.64 to 0.78, 0.91 to 0.95, 0.58 to 0.84, and 0.69 to 0.80 (Fig. 1).

#### Reliability analysis

Reliability analysis was also conducted on the base of 441 participants. Cronbach’s alpha coefficient was calculated to determine the internal consistency of the C-PPRS. Results indicated that C-PPRS and the four subscales (understanding, intimacy, acceptance, and trust) all had adequate internal consistencies, with Cronbach’s alpha coefficients were 0.90, 0.81, 0.92, 0.78, and 0.81, respectively.

## Study 2

### Method

#### Participants

The participants were recruited via online data collecting platforms (i.e., https://www.wjx.cn). A total of 253 subjects completed the online questionnaire. The inclusion and exclusion criteria were the same as study1, 29 participants were excluded, and 224 valid participants were obtained with an effective rate of 88.54%. Detailed demographic information is shown in Table 1. The average age of the participants was 24.00 (*SD* = 6.05). Among them, 21.9% were male, and 78.1% were female. 26 participants (11.6%) were married, and 198 participants (88.4%) were currently in a romantic relationship. Their average duration of the relationship was 3.11 (*SD* = 4.30).

#### Measures

*Demographic Information* The demographic questionnaire included age, gender, marital status (married or in a romantic relationship), and duration of the relationship.

*C-PPRS* The C-PPRS used in this study was the 15-item scale developed in study 1. All the items were rated on a 5-point Likert scale (1 = totally disagree, 5 = totally agree). The higher the scale score was, the higher the level of responsiveness that one perceives from the partner was. The Cronbach’s alpha coefficients of four factors and the whole scale in this sample were 0.78, 0.88, 0.75, 0.74, and 0.87, respectively.

*The Quality of Relationship Index (QRI)* The QRI, a 6-item scale developed by Patrick et al. [15], was widely used to measure relationship satisfaction (e.g., “My relationship with my partner makes me happy”). All the items were rated on a 7-point Likert scale (1 = totally disagree, 7 = totally agree). The Chinese version of the QRI has been demonstrated to be a reliable and valid measurement in assessing relationship satisfaction among the Chinese [20, 21]. The Cronbach’s alpha of the scale in this sample was 0.92.

### Results

#### Concurrent validity

The concurrent validity was analyzed by calculating the correlation coefficient between the C-PPRS score and the QRI score. The correlation coefficients of the four subscales and QRI were 0.61, 0.30, 0.48, and 0.63, respectively, and the *p* values were all below 0.001. A moderate but statistically significant correlation was found between the total score of C-PPRS and QRI (*r* = 0.66 *p* < 0.001), demonstrating an excellent concurrent validity.

## Discussion

The main purpose of the present study was to develop a psychometrically robust instrument to assess PPR in the Chinese population, based on the theoretical framework of the previous English version [12] and the interview results of the pilot study. This study described the development and validation of the C-PPRS as an instrument to assess PPR using 15 items as indicators of the four responsiveness components (i.e., understanding, intimacy, acceptance, and trust).

C-PPRS was compiled and revised locally in our two studies, taking subjects of different ages (all over 18 years old) and different relationship durations as research samples. In addition, the factor structure (analyzed by EFA and CFA), reliability, and concurrent validity were examined.

In the first study, an EFA was conducted in Sample A to explore the factor structure of the 15 items that comprised the C-PPRS. Results suggested a structure of four factors that reflected the core components of PPR. The factor loadings of items were between 0.60 and 0.93. The cumulative variance explanation rate reached 68.53%. This structure was also confirmed in Sample B, using a CFA providing evidence for the four-factor structure. The factor loadings were between 0.58 and 0.95. The results demonstrated that the four-factor model was better fitted than the single-factor structure. The C-PPRS showed good factorial structure, and there were consistent outcomes across the two samples. All four components and the total composite score of C-PPRS showed good internal reliability in study 1. Cronbach’s alpha coefficients above 0.70 are usually indications of a reliable set of items [19]. In our study, the whole scale and all the factors were above the 0.70 cut-off score, ranging between 0.78 and 0.92.

PPR is an essential element of satisfying romantic relationships, especially insofar as it facilitates and enhances relationship quality. PPR is a robust positive predictor of relationship quality, as shown in cross-sectional, longitudinal and laboratory studies [22–24]. For example, A study of 3593 participants from 57 countries during the COVID epidemic showed a significant positive correlation between PPR and relationships quality (*r* = 0.63) [22]. Crocker’s study indicated that PPR predicted the quality of the romantic couple’s relationship (*r* = 0.32) [23]. Laboratory experiments also showed that partners’ responsiveness in episodic memory was significantly positively related to actors’ QRI (*r* = 0.38) [24]. Concurrent validity was examined in Study 2, verifying the relationships of C-PPRS subscales, the total scores, and the QRI. All four components and the total scores of the C-PPRS were positively correlated with the score of the QRI. In other words, the results showed that there was a significantly positive correlation between PPR and relationship satisfaction. This is consistent with previous research, which suggested that when individuals perceive their partner’s response, they feel understood, cared for, accepted and trusted, leading to increased intimacy and satisfaction in the relationship [25]. In general, the C-PPRS was shown to be extremely related to a similar construct, which provided further support to the robustness of the instrument’s validity.

These results provided support for the robust psychometric characteristics of the C-PPRS. In light of this, the C-PPRS appears to have the necessary characteristics to adequately assess PPR overcoming the limitations of the previous instruments.

The C-PPRS was developed to create three or four items for each component. The number of items is ideal because it allows the opportunity to conduct structural equation modeling analyses with latent variables (a minimum of three indicators per latent construct is generally recommended) [19]. Also, it is concise enough to be used in combination with other psychometric instruments.

### Theoretical and practical implications

The findings of this study contribute to the literature on intimate relationships. Intimacy is an interpersonal interaction process, and if individuals want to experience intimate interactions with their partners, individuals need to perceive their partners’ responses, so PPR has a crucial role in the interaction between partners. Therefore, assessing and capturing individuals’ PPR in their interactions with their partners becomes crucial to advancing research in the field of intimacy. Based on Reis et al.’s theoretical framework and our pilot study, this study developed and tested a four-factor instrument applicable to Chinese adults’ assessment of romantic relationship partners rather than friends in general. Building on Reis’ two-dimensional scale [8], this study combines Reis et al.’s description of four aspects of PPR [14] to propose a four-dimensional framework for measuring PPR. This study argues that individuals need to perceive four aspects of their partners’ responses-understanding, intimacy, acceptance, and trust-if they are to experience intimate interactions in their relationships, which makes an important contribution to the literature.

The findings of this study have important practical implications for the maintenance or development of relationships for individuals in intimate relationships or marriages. This study found a positive correlation between PPR and relationship satisfaction, suggesting that PPR may reflect the quality of intimacy to some extent. The result implies that improving PPR may be an intervention that can improve relationship satisfaction for both partners. For instance, individuals who are in an intimate relationship or who are married can improve their PPR to maintain relationship satisfaction; counselors can enhance their PPR by assigning tasks that lead them to feel the partner’s response or actively give the partner a response in order to improve their PPR and thus enhance relationship satisfaction.

### Limitations and future research

The present study has several limitations that need to be addressed in future research despite obtaining interesting results. First, PPR in the present study was assessed using a self-reported measure that can be influenced by social desirability and other common method biases. Future research could use qualitative interviews, diary studies, and/or other methods to replicate these findings. Second, it is shown that PPR was significantly correlated with Arabian women’s sexual function [7]. Similarly, the Chinese often feel ashamed when talking about sex. If PPR measured by C-PPRS can also reflect sexual quality in Chinese couples, it can be helpful in Chinese sex-related studies. Therefore, further research is needed to examine whether C-PPRS can detect and assess the partner with sexuality. Finally, our study used a cross-sectional design, which cannot provide evidence for a causal relationship between the instrument and the criteria. Future research should include longitudinal designs to provide more convincing evidence to assess test–retest reliability and predictive validity.

## Conclusion

In general, the C-PPRS measures the individual PPR from four dimensions (understanding, intimacy, acceptance, and trust, containing 4, 3, 4, and 4 items, respectively). The scale is concise, with clear and lucid expressions. It has appropriate psychometric properties regarding structural validity, internal consistency reliability, and concurrent validity when tested in Chinese.

## Supplementary Information

**Additional file 1.** Initial Items of the C-PPRS.

## Data Availability

The datasets used and/or analyzed during the current study are available from the corresponding author on reasonable request.
