# Supplementary material for: Development and validation of the Chinese version of the perceived partner responsiveness scale (C-PPRS)
Source: BMC Psychol. 2022 Jun 20;10:155. doi: 10.1186/s40359-022-00865-x (PMC9208143; doi:10.1186/s40359-022-00865-x)
Supplement: Supplementary file 1 — Additional file 1. Initial Items of the C-PPRS. [file 40359_2022_865_MOESM1_ESM.docx]

**Initial Items of the Chinese Version of the Perceived Partner Responsiveness Scale (C-PPRS)**

Understanding

1. My partner understands my fear of something.

2. My partner understands what kind of life I want.^*^

3. My partner can stand on my feet and understand my feeling.^*^

4. My partner understands what I prefer or hate.^*^

5. My partner can understand why I am sometimes angry.

6. My partner is usually clear about what my decision is based on.^*^

7. My partner knows why I sometimes cannot respond to him or her.

8. My partner understands why I like or dislike something.

Intimacy

1. My partner is willing to be intimate with me.

2. My partner often expresses how much he or she misses me.^*^

3. When my partner is not by my side, he or she will tell me that he or she misses me.^*^

4. My partner often expresses his or her love to me.^*^

5. My partner will give me a hand if I am in trouble.

6. My partner usually shares daily life with me.

7. My partner always prepares gifts for me during the anniversary.

8. My partner will give me a sense of security even if he or she is not by my side.

Acceptance

1. My partner accepts my bad side.^*^

2. My partner thinks that I don’t need to make changes for him or her.^*^

3. My partner seldom blames me blindly for my mistakes.^*^

4. My partner embraces my flaws.^*^

5. My partner can tolerate my bad temper.

6. My partner doesn’t mind my previous romantic relationship excessively.

7. My partner doesn’t mind my background and social status.

8. My partner accepts my different hobbies.

Trust

1. My partner thinks that I am responsible for our relationship and family.^*^

2. My partner believes that I am good and reliable.^*^

3. My partner doesn’t mind showing his disadvantages to me.

4. My partner considers that our relationship is strong.^*^

5. My partner doesn’t mind telling secrets to me.

6. My partner does not always interfere with my social circle.

7. My partner always considers my thoughts when making important decisions.

8. My partner always believes me when I am misunderstood by others.^*^

*Note*: Items marked with an asterisk (*) are those included in the final version of C-PPRS.
